# Neuropathic pain experience in symptomatic and presymptomatic subjects carrying a transthyretin gene mutation

**DOI:** 10.3389/fneur.2023.1109782

**Published:** 2023-02-08

**Authors:** Stefano Tozza, Marco Luigetti, Giovanni Antonini, Anna Mazzeo, Daniele Severi, Andrea Di Paolantonio, Luca Leonardi, Massimo Russo, Angela Romano, Francesca Forcina, Luca Gentile, Maria Nolano, Consalvo Mattia, Fiore Manganelli

**Affiliations:** ^1^Department of Neuroscience, Reproductive Sciences and Odontostomatology, University of Naples “Federico II”, Naples, Italy; ^2^Fondazione Policlinico Universitario A. Gemelli IRCCS, UOC Neurologia, Rome, Italy; ^3^Department of Neuroscience, Università Cattolica del Sacro Cuore, Rome, Italy; ^4^Department of Neuroscience, Mental Health and Sensory Organs (NESMOS), Sapienza University, Rome, Italy; ^5^Unit of Neurology and Neuromuscular Diseases, Department of Clinical and Experimental Medicine, University of Messina, Messina, Italy; ^6^U.O. Neurologia, Fondazione Poliambulanza Istituto Ospedaliero, Brescia, Italy; ^7^Neurology Department, Skin Biopsy Laboratory, Istituti Clinici Scientifici Maugeri IRCCS, Telese Terme, Italy; ^8^Department of Medical and Surgical Sciences and Biotechnologies, Faculty of Pharmacy and Medicine, “Sapienza” University of Rome, Rome, Italy; ^9^Anesthesia, Intensive Care and Pain Unit, ICOT-Polo Pontino, Latina, Italy

**Keywords:** ATTRv, hereditary amyloidosis, pain, quality of life, transthyretin

## Abstract

**Introduction:**

Pain is a common symptom of hereditary transthyretin amyloidosis (ATTRv), however, its occurrence in late-onset ATTRv has not been investigated thoroughly. Our aim was to describe the pain experience and its impact on quality of life (QoL) in symptomatic patients and presymptomatic carriers harboring a transthyretin (*TTR*) gene mutation with a late-onset phenotype.

**Materials and methods:**

Study participants (aged ≥18 years) were consecutively recruited from four Italian centers. Clinical disability was assessed using the Familial Amyloid Polyneuropathy (FAP) stage and Neuropathy Impairment Score (NIS). The Norfolk questionnaire evaluated QoL and the Compound Autonomic Dysfunction Test assessed autonomic involvement. Neuropathic pain was screened using the Douleur Neuropathique 4 (DN4) questionnaire, and pain intensity and its impact on daily activity were assessed using the Brief Pain Inventory severity and interference subscores. Data on the type of *TTR* mutation, presence of cardiomyopathy, treatment, and Body Mass Index (BMI) were collected.

**Results:**

Overall, 102 subjects with *TTR* mutations (mean age ± SD 63.6 ± 13.5 years) were recruited, including 78 symptomatic patients (68.1 ± 10.9 years) and 24 presymptomatic carriers (49 ± 10.3 years). Pain was reported by 75.5% of all subjects, but was more frequent in symptomatic patients than in presymptomatic carriers (85.9 vs. 41.6%, respectively). Pain exhibited neuropathic features (DN4≥4) in 69.2% of symptomatic patients and in 8.3% of presymptomatic carriers. Subjects with neuropathic pain were older (*p* = 0.015) had worse FAP stage (*p* < 0.001), higher NIS scores (*p* < 0.001), greater autonomic involvement (*p* = 0.003), and a lower QoL (*p* < 0.001) than those without neuropathic pain. Neuropathic pain was associated with higher pain severity (*p* < 0.001) and had a significant negative impact on daily activities (*p* < 0.001) Neuropathic pain was not associated with gender, mutation type, TTR therapy, or BMI.

**Conclusion:**

Approximately 70% of late-onset ATTRv patients complained of neuropathic pain (DN4≥4) that worsened as peripheral neuropathy progressed and increasingly interfered with daily activities and QoL. Notably, 8% of presymptomatic carriers complained of neuropathic pain. These results suggest that assessment of neuropathic pain may be useful to monitor disease progression and identify early manifestations of ATTRv.

## 1. Introduction

Hereditary transthyretin amyloidosis (ATTRv; v for “variant”) is a progressive multisystem disorder caused by mutations in the *transthyretin* (*TTR*) gene. The mutated TTR tetramer dissociates in misfolded monomers that accumulate in tissues such as the heart and peripheral nervous system (PNS), leading, respectively, to cardiomyopathy and progressive axonal peripheral neuropathy (1). PNS involvement is the presenting complaint in most cases of ATTRv and causes an axonal length-dependent sensory-motor and autonomic polyneuropathy (2). According to the type of mutation and geographic area, two different ATTRv phenotypes exist (3): the early-onset phenotype ( ≤ 50 years) has a predominant small fiber involvement and is typical in endemic regions, while the late-onset phenotype (>50 years), which is typical of non-endemic areas such as Italy (4), has a progressive and prevalent large fiber damage (5).

Several symptoms are reported by patients. The most frequent symptoms are pain, numbness, fatigue, and weakness (6) that substantially impact on the functioning, well-being, and quality of life (QoL) of patients (7) and their relatives (8). Notably, health status is severely impaired by pain/discomfort in 70% of ATTRv patients (9).

Pain, including electric shock, tingling, pins and needles, burning, and itching sensations (10), is widely documented in the early-onset ATTRv phenotype, whereas it has not been thoroughly investigated in the late-onset phenotype. In fact, in the early-onset phenotype, in keeping with the preferential loss of small nerve fibers (11), the patients primarily notice pain and thermal sensation dysfunction as well as dysautonomia. Conversely, in late-onset ATTRv phenotype, the small nerve fiber involvement may remain under recognized if not properly investigated, as it is usually overwhelmed by motor and sensory impairment.

Consequently, the aim of this study was to describe the neuropathic pain experience, its frequency, and impact on QoL in symptomatic patients and presymptomatic carriers harboring a *TTR* gene mutation associated with the late-onset ATTRv phenotype.

## 2. Material and methods

This multicenter study was conducted in four Italian centers with experience in the diagnosis and management of ATTRv (University of Naples Federico II, Fondazione Policlinico Universitario A. Gemelli IRCCS of Rome, Sant'Andrea Hospital of Sapienza University of Rome, University of Messina). The study was approved by the local institutional ethics committee in each center.

Study participants (aged ≥18 years) were consecutively recruited and included both symptomatic patients and presymptomatic carriers with known pathogenic *TTR* mutations. Carriers, who had normal nerve conduction, were identified as carrying a *TTR* gene mutation in the setting of genetic familial counseling (12).

Subjects were excluded from study if they had concomitant disease (e.g., diabetes) in which pain could represent a relevant feature.

Age at enrolment, gender, mutation type, ATTRv therapy, pain killers, body mass index (BMI), and the presence of cardiomyopathy were recorded.

Symptomatic patients and presymptomatic carriers underwent an extensive neurological evaluation, and several scales were applied to assess disease stage (Familial Amyloid Polyneuropathy [FAP] stage) (13), neuropathic impairment (Neuropathy Impairment Score [NIS]) (14), autonomic involvement (Compound Autonomic Dysfunction Test [CADT]) (15), and QoL (Norfolk Quality of Life-Diabetic Neuropathy [QOL-DN]) (16).

The presence of pain and its distribution (generalized, distal at upper and lower limbs, at lower limbs or at upper limbs) was recorded. The Douleur Neuropathique 4 (DN4) questionnaire was used to establish if pain had neuropathic features (DN4 ≥4) (17). The Brief Pain Inventory (BPI) evaluated the severity of pain (BPI severity) and its impact on daily activity (BPI interference) (18).

### 2.1. Statistical analysis

Descriptive analyses were based on the mean±standard deviation (SD) for numerical variables and percentage for categorical data. After verifying the Gaussian distribution of variables through the Kolmogorov–Smirnov test, the Student's *T*-test was used to compare continuous variables between subjects with neuropathic pain (DN4 ≥4) and those without neuropathic pain (DN4 < 4). Pearson's chi-square test was used to compare categorical variables between these groups. When significant differences were detected (*p* < 0.05), *post-hoc* analyses of categorical variables through adjusted standardized residuals with Bonferroni-corrected *p-*values (*p* ≤ 0.006) were calculated in order to identify which cells of the contingency table contributed most to the significant effect (19). Analyses and graphics were performed using the statistical software IBM SPSS Statistic version 25.

## 3. Results

In total, 102 subjects with *TTR* mutations (mean age ± SD 63.6 ± 13.5 years) were recruited, including 78 symptomatic patients (68.1 ± 10.9 years) and 24 presymptomatic carriers (49 ± 10.3 years). Most symptomatic patients had a disease severity of FAP 1 (sensory neuropathy) or FAP 2 (require assistance for walking). Demographic and clinical findings are summarized in Table 1.

**Table 1:** Demographic and clinical findings.

	**Overall *N* = 102**	**Symptomatic patients *N* = 78**	**Presymptomatic carriers *N* = 24**
**Age at enrolment, years**	63.6 ± 13.5 (30–86)	68.1 ± 10.9 (31–86)	49 ± 10.3 (30–77)
**Mutation**			
Val30Met	43.1%	38.5%	45.8%
Phe64Leu	37.3%	42.3%	33.4%
Others	19.6%	19.2%	20.8%
**Gender (Male/Female)**	72/30	59/19	13/11
**FAP stage**			
0	23.5%	–	100%
1	43.1%	56.4%	–
2	31.4%	41.0%	–
3	2.0%	2.6%	–
**BMI**	25.1 ± 4.5 (16–40)	24.7 ± 4.5 (16–40)	26 ± 4.4 (19–37)
**NIS**	49.8 ± 50.1 (0–173)	61.4 ± 49.1 (0–173)	1 ± 1.5 (0–6)
**CADT**	15.9 ± 3.4 (6–20)	15 ± 3.3 (6–20)	18 ± 2 (15–20)
Male	16.2 ± 3.6 (6–20)	15.4 ± 3.5 (6–20)	19.8 ± 0.3 (19–20)
Female	14.6 ± 2 (10–16)	13.9 ± 2.2 (10–16)	15.9 ± 0.3 (15–16)
**Norfolk QOL-DN**	42.7 ± 34.9 (-2–120)	53.9 ± 32.1 (-1–120)	9.2 ± 9.7 (-2–33)
**Pain**	75.5% (77/102)	85.9% (67/78)	41.6% (10/24)
**Distribution of pain**			
Generalized	29.9% (23/77)	32.8% (22/67)	10% (1/10)
Distal	44.1% (34/77)	46.4% (31/67)	30% (3/10)
Lower limbs	13.0% (10/77)	10.4% (7/67)	30% (3/10)
Upper limbs	13.0% (10/77)	10.4% (7/67)	30% (3/10)
**Neuropathic pain** (DN4≥4)	54.9% (56/102)	69.2% (54/78)	8.3% (2/24)
**Numeric rating scale for pain (BPI_3)**	4.6 ± 3.5 (0–10)	5.4 ± 3.2 (0–10)	1.9 ± 2.9 (0–9)
**BPI severity**	3.3 ± 2.7 (0–10)	43.9 ± 2.5 (0–10)	1.1 ± 1.9 (0–7)
**BPI interference**	3.2 ± 2.9 (0–9.7)	3.9 ± 2.8 (0–9.7)	0.8 ± 1.5 (0–6.4)
**Presence of cardiomyopathy**	50%	65.4%	0%
**TTR therapy**			
Tafamidis	19.6%	25.6%	–
Patisiran	23.6%	30.8%	–
Inotersen	13.7%	17.9%	–
Liver transplant	0.9%	1.4%	–
None	42.2%	24.3%	100%

Data are shown as mean ± standard deviation (range) or percentage.

BMI, Body Mass Index; BPI, Brief Pain Inventory; CADT, Compound Autonomic Dysfunction Test; FAP, Familial Amyloid Polyneuropathy; NIS, Neuropathy Impairment Score; QOL-DN, Quality of Life-Diabetic Neuropathy; TTR, Transthyretin.

Pain was reported by 75.5% of all subjects, but was more frequent in symptomatic patients than in presymptomatic carriers (85.9 vs. 41.6%, respectively). The distribution of pain was predominantly generalized or distal in symptomatic patients, whereas presymptomatic carriers reported distal pain or pain in the lower or upper limbs. Neuropathic pain (DN4 ≥4) was reported by 69.2% of symptomatic patients and 8.3% of presymptomatic carriers. These patients reported more frequently electric shock (66.7%) and less frequently burning (54.3%) and painful cold (52.6%) sensation. The pain was typically associated with tingling and numbness (91.2%) and less frequently with pins and needles (63.2%) and itching (31.6%) sensation. Reduced touch and pinprick sensation was present in the 82.5 and 68.4% respectively, whereas the allodynia phenomenon was reported only in the 49.1% of patients suffering from neuropathic pain.

**Figure 1:**
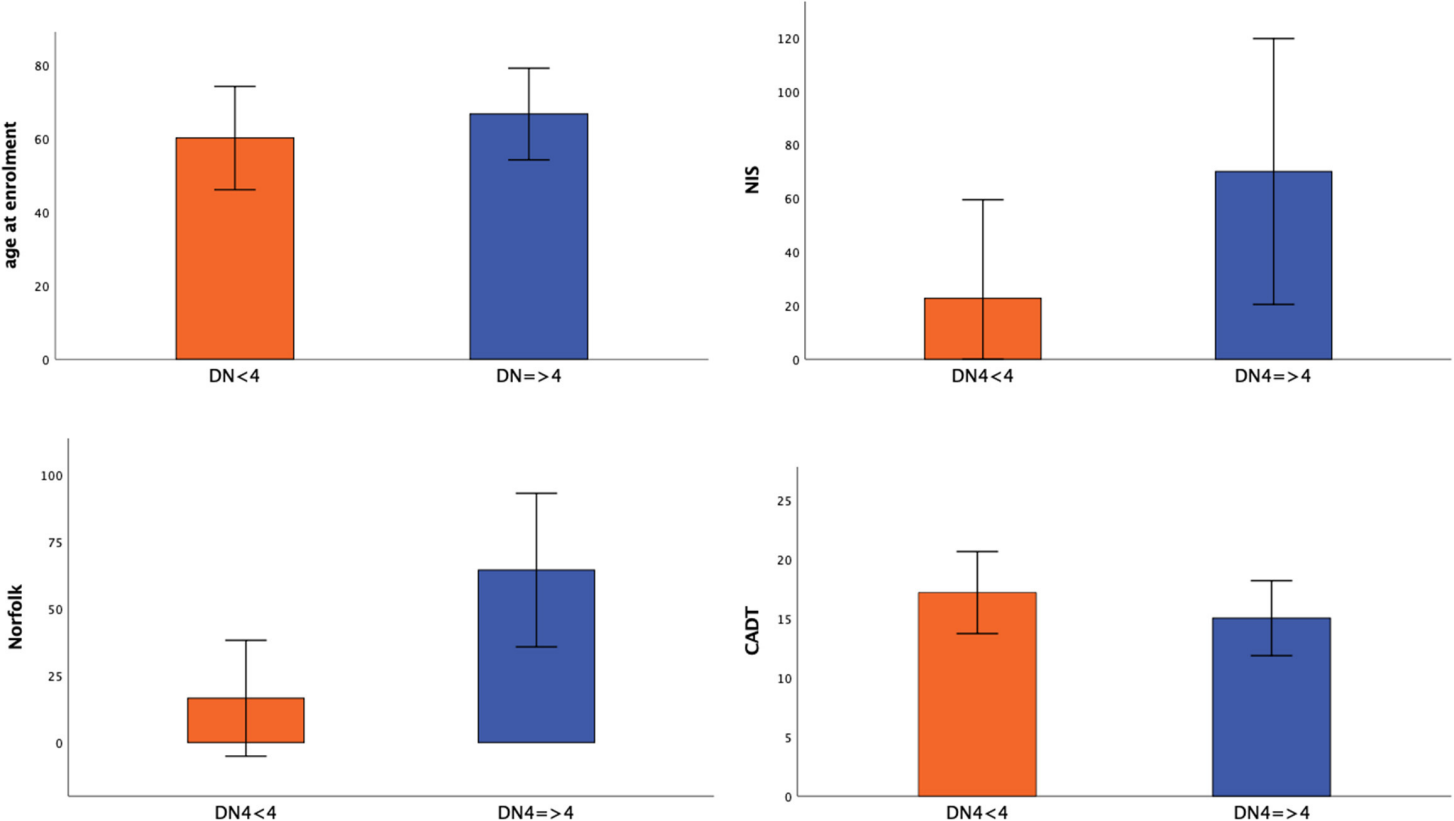
Significance differences between patients without (DN4 < 4) and with neuropathic pain (DN4 > 4). Mean value ± standard deviation of age at enrolment, NIS, Norfolk, CADT between patients without (DN4 < 4) and with neuropathic pain (DN4 > 4) was represented.

Subjects with neuropathic pain were significantly older (66.5 vs. 60.1 years; *p* = 0.015) and had more severe disease (FAP 1–2 vs. 0–1, *p* < 0.001) and neuropathic impairment (NIS, 69.9 ± 49.5 vs. 22.6 ± 36.8; *p* < 0.001) than subjects without neuropathic pain (DN4 < 4). The presence of neuropathic pain was associated with significantly greater autonomic involvement (CADT; *p* = 0.003) and lower QoL (Norfolk QOL-DN; *p* < 0.001) compared with no neuropathic pain (Figure 1).

**Figure 2:**
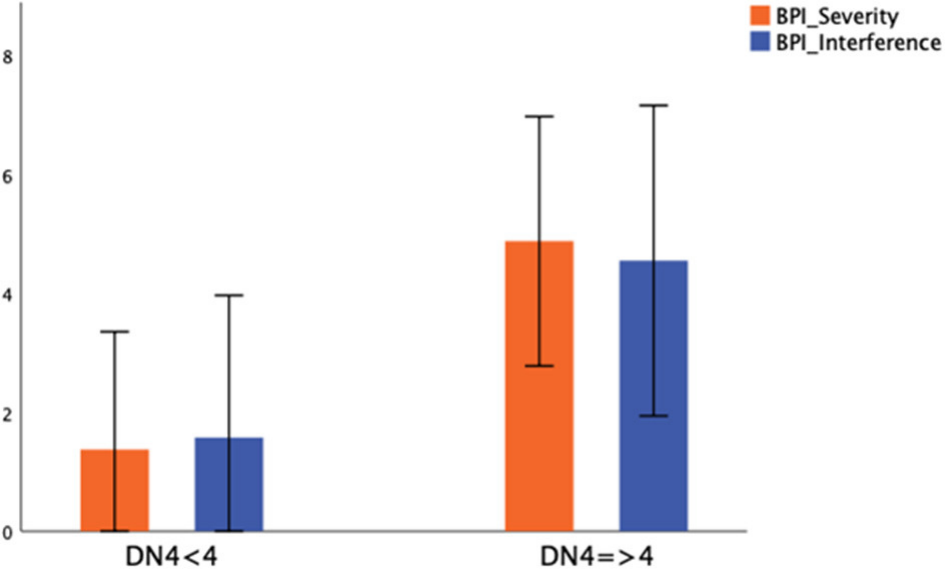
Severity and daily life interference in patients without (DN4 < 4) and with neuropathic pain (DN4 > 4). Mean value ± standard deviation of BPI Severity and BPI Interference between patients without (DN4 < 4) and with neuropathic pain (DN4 > 4) was represented.

Significantly more subjects with neuropathic pain had cardiomyopathy than those without neuropathic pain (*p* = 0.005) (Table 2). The presence of neuropathic pain was associated with significantly higher pain severity (BPI severity; *p* < 0.001) and had a significant negative impact on daily activities (BPI interference; *p* < 0.001) (Figure 2).

**Table 2:** Comparison of demographic and clinical data.

	**DN4 < 4 *N* = 46**	**DN4≥4 *N* = 56**	***p-value***
**Age at enrolment, years**	60.1 ± 14	66.5 ± 12.4	**0.015**
**Gender**			
Male	31	41	0.335
Female	15	15	
**Mutation**			
Val30Met	22	21	0.535
Phe64Leu	17	22	
Others	7	13	
**BMI**	25.7 ± 4.5	24.4 ± 4.4	0.208
**FAP stage**			
0	22^*^	2^*^	**< 0.001**
1	21	23	
2	2^*^	30^*^	
3	1	1	
**NIS**	22.6 ± 36.8	69.9 ± 49.5	**< 0.001**
**CADT**	16.8 ± 3.3	14.8 ± 3	**0.003**
**Norfolk QOL-DN**	16.4 ± 21.6	64.2 ± 28.6	**< 0.001**
**BPI severity**	1.3 ± 1.9	4.8 ± 2.1	**< 0.001**
**BPI interference**	1.6 ± 2.4	4.5 ± 2.6	**< 0.001**
**Cardiomyopathy**			
No	30	21	**0.005**
Yes	16	35	
**TTR Therapy**			
Tafamidis	9	11	0.09
Patisiran	5	19	
Inotersen	2	12	

^*^Indicates significance (p ≤ 0.006) for the post-hoc analyses. Data are shown as mean ± standard deviation or number.

Bold value indicates a statistically significant difference with a p-value less than 0.05.

BMI, Body Mass Index; BPI, Brief Pain Inventory; CADT, Compound Autonomic Dysfunction Test; FAP, Familial Amyloid Polyneuropathy; NIS, Neuropathy Impairment Score; QOL-DN, Quality of Life-Diabetic Neuropathy; TTR, Transthyretin.

Neuropathic pain was not associated with gender, mutation type, TTR therapy, or BMI (Table 2).

Neuropathic pain was treated in 82.4% of subjects, most frequently with anti-seizure drugs (63.8%) like pregabalin or gabapentin. Seven subjects were treated with NSAIDs, 3 with opioids, 1 with antidepressants, 1 with local steroidal injection, and 4 with combination therapy. Patients reported a satisfactory pain relief (>50% on the item BPI_8) in less than half of cases (45.9%), whereas 40.5% of patients referred persistent pain despite the therapy (< 30% on the item BPI_8).

## 4. Discussion

Pain is a common symptom in ATTRv (6) and impacts patients' functioning, health status, and QoL (7). However, the frequency of neuropathic pain in the late-onset ATTRv phenotype is unknown.

To our knowledge, this is the first systematic assessment of pain in a large cohort of late-onset *TTR*-mutated subjects encompassing both symptomatic patients and presymptomatic carriers. We showed here that ~70% of symptomatic patients experienced neuropathic pain (DN4 ≥4) with a length-dependent distribution. The pain was mainly characterized by electric shock associated with tingling, numbness and reduced touch sensation. All these features are consistent with a prominent involvement of larger myelinated A-β fibers (20) as typically occurs in late-onset ATTRv (3).

When compared with subjects without neuropathic pain, those with neuropathic pain were older, exhibited a more severe disability (FAP stage and NIS), had a lower QoL (Norfolk questionnaire), had significant autonomic involvement (CADT), and were more likely to have cardiomyopathy. Accordingly, we propose that neuropathic pain in ATTRv worsens as disease progresses and increasingly impacts patients' QoL.

This suggests that a thorough assessment of pain is important to define an optimal treatment regimen. In addition, careful pain investigation may offer relevant information to clinicians for monitoring disease worsening or to assess multisystem involvement (e.g., cardiomyopathy).

Interestingly, we found that 8% (*N* = 2) of presymptomatic carriers reported neuropathic pain. Both subjects were young women (aged 33 and 40 years at enrolment) and carriers of the Glu89Gln variant. Neither reported dysautonomic (CADT=16) or neuropathic (NIS= 0) symptoms, whereas both complained of a mild impact in QoL (Norfolk = 33 and 10).

This finding raises the question of whether presymptomatic carriers who complain of neuropathic pain should instead be considered symptomatic patients and, consequently, be able to access the available therapies. Indeed, according to expert consensus (12), neuropathic pain, including spontaneous and evoked symptoms, could be considered as definitively related to the onset of symptomatic ATTR amyloidosis even without an instrumental demonstration of small nerve fiber impairment (e.g., quantitative sensory testing, skin biopsy). Nonetheless, the finding of pain, especially if neuropathic, should lead to further assessment for neuropathy beyond the conventional nerve conduction study.

The results of our study, therefore, suggest that it would be judicious to query the presence of neuropathic pain in presymptomatic relatives who carry a *TTR* gene mutation, as its presence might represent an early manifestation of ATTRv. In this perspective, it is largely documented that small nerve fiber damage (21–23) may precede, by many years, the signs and symptoms of neuropathy in the late-onset ATTRv phenotype.

Lastly, we found that almost one fifth of patients (17.6%) had not received any symptomatic treatment for neuropathic pain. Moreover, patients receiving painkillers did not achieve significant pain relief in about 40% of the cases. In the late-onset ATTRv phenotype, pain and its treatment could be overlooked if clinicians are more focused on motor disability.

## 5. Conclusion

In conclusion, clinicians should not neglect the pain experience when evaluating ATTRv patients or presymptomatic relatives who carry a *TTR* gene mutation. The DN4 questionnaire is a reliable screening tool to identify, with high specificity, the presence of neuropathic pain (17). As pain impacts QoL, individuals who experience pain have the right to appropriate pain relief. Assessment of neuropathic pain may be a useful strategy to monitor disease progression in symptomatic patients and may be suitable to identify early manifestation of the disease in presymptomatic relatives who carry a *TTR* gene mutation.

## Data availability statement

The raw data supporting the conclusions of this article will be made available by the authors, without undue reservation.

## Ethics statement

The studies involving human participants were reviewed and approved by Local Ethical Board University of Naples Federico II of Naples. The patients/participants provided their written informed consent to participate in this study.

## Author contributions

ST contributed to the acquisition, analysis and interpretation of data for the work, drafted, and revised the work. ML, GA, AM, and MN contributed to the acquisition, interpretation of data for the work, and revised the work. DS, AD, LL, MR, AR, FF, and LG contributed to the acquisition of data for the work and revised the work. CM contributed to the conception and design of the work and revised the work. FM contributed to the conception and design of the work, interpretation of data for the work, and drafted and revised the work. All authors gave final approval for the final version to be published and agreed to be accountable for all aspects of the work in ensuring that questions related to the accuracy or integrity of any part of the work are appropriately investigated and resolved.
